# Effect of guanidine acetic acid on meat quality, muscle amino acids, and fatty acids in Tibetan pigs

**DOI:** 10.3389/fvets.2022.998956

**Published:** 2022-10-11

**Authors:** Yiyan Cui, Zhimei Tian, Miao Yu, Zhichang Liu, Ting Rong, Xianyong Ma

**Affiliations:** ^1^Institute of Animal Science, Guangdong Academy of Agricultural Sciences, Guangzhou, China; ^2^State Key Laboratory of Livestock and Poultry Breeding, Guangzhou, China; ^3^The Key Laboratory of Animal Nutrition and Feed Science in South China, Ministry of Agriculture, Guangzhou, China; ^4^Guangdong Provincial Key Laboratory of Animal Breeding and Nutrition, Guangzhou, China; ^5^Guangdong Engineering Technology Research Center of Animal Meat Quality and Safety Control and Evaluation, Guangzhou, China; ^6^Maoming Branch, Guangdong Laboratory for Lingnan Modern Agriculture, Maoming, China

**Keywords:** guanidine acetic acid, fatty acid, lipid metabolism, Tibetan pig, meat quality

## Abstract

This study investigated the effects of guanidine acetic acid (GAA) supplementation on growth performance, carcass traits, and meat quality in Tibetan pigs. A total of 18 male Tibetan pigs (21.35 ± 0.99 kg) were randomly assigned to the control (basal diet) and GAA (basal diet + 800 mg/kg GAA) groups for 125 days. Growth performance, carcass traits, and meat quality in pigs, and the chemical composition of *Longissimus thoracis* (LT) were not altered by GAA. In LT, compared to the control group, dietary GAA increased the superoxide dismutase activity, transcripts of *stearoyl CoA desaturase* (*SCD*) and *fatty acid synthase* (*FAS*), and contents of glutamate, glutamine, C24:0, C20:3n-6, C20:4n-6, and polyunsaturated fatty acids (*P* < 0.05), but it decreased the malondialdehyde content (*P* < 0.001). In back fat, dietary GAA reduced the transcript of peroxisome proliferator-activated receptor γ (*PPAR*γ) and the contents of C10:0, C12:0, C14:0, and C16:0 (*P* < 0.05), whereas it increased the contents of C22:0, C20:1, C22:1, C24:1, C20:2, C20:3n-3, and C22:2 (*P* < 0.05). These findings will provide a basis for high-quality Tibetan pork production.

## Introduction

Commercial pigs have greater weight and lean meat production, but have significantly lower intramuscular fat (IMF) content, which has a negative impact on tenderness, juiciness, color, and flavor (1). Consumers are gravitating toward safer, healthier, and tastier meats. Local pig breeds are resources that can meet the diversified needs of consumers. Chinese local pig breeds are better accepted by Chinese consumers because of their dark red meat color, high fat content, and good flavor. Tibetan pigs live on the Qinghai-Tibet Plateau and are one of the local pig breeds in China. They have characteristics, such as coarse feed resistance, fine and tender meat, strong disease resistance, and strong adaptability to the environment. However, Tibetan pigs have lower potential for growth and protein deposition (low meat production) compared to modern pig breeds (2). Hence, a question arises as to how to increase the Tibetan pig meat production? Nutritional strategy is one of the most common methods to improve pork production.

Guanidine acetic acid (GAA) is the precursor of creatine, which is synthesized by arginine and glycine in the kidney, liver, and pancreas; methylated to creatine and then involved in energy and protein metabolism (3). *In vitro* experiments have shown that GAA could promote the rate of fusion of myotubes in C2C12 myoblasts and increase the thickness of myotubes and cross-sectional area of the gastrocnemius muscle (4). GAA supplementation in the diet helps to improve growth performance, muscle yield, and meat quality of broilers and pigs (5–9). GAA not only increased the weight of skeletal muscle and up-regulated the expression of myosin heavy chain (MyHC) I mRNA in skeletal muscle, but it also decreased the cross-sectional area and diameter of muscle fiber (10, 11). Jayaraman et al. (7) reported that the diet supplemented with GAA couldimprove the lean meat yield of finishing pigs. It can be seen from the above studies that the research on GAA has mainly focused on muscle fibers, lean mass, and skeletal muscle development, and especially in commercial pigs. However, few studies have been performed in Tibetan pigs, and little is known about the effects of GAA on Tibetan pigs. Arginine and creatine are related metabolites of GAA, and GAA supplementation can indirectly play their role (12). Arginine alters the fatty acid content and composition in tissues (13). At present, only one study has mentioned the effect of GAA on fatty acids in grass carp (14). Tibetan pig is a miniature pig breed with a high lipid deposition characteristic (15). Therefore, we speculated that GAA will change the fatty acid profile of muscle and adipose tissues in Tibetan pigs. In short, the regulatory effects of dietary GAA on growth performance, meat quality, and fatty acid profile of Tibetan pigs have not been studied. This study compared the carcass traits, meat quality, free amino acid contents, and fatty acid contents of Tibetan pigs with or without GAA supplementation, in order to provide a reference for better production of Tibetan pigs.

## Materials and methods

### Animals and diets

Animal procedures and experiments were approved by the Animal Care and Use Committee of Guangdong Academy of Agricultural Sciences (authorization number GAASIAS-2019-0806).

A total of 18 male Tibetan pigs (initial weight 21.35 ± 0.99 kg) were randomly assigned to the control and GAA groups. Each treatment comprised 9 replicate pens, with 1 barrow in each. The experimental period lasted 125 days. The control group was fed a basal diet, and the GAA group was fed a basal diet supplemented with 800 mg/kg GAA. We use commercial feed (Guangda 332, Guangdong Guangda Feed Co., Ltd., Guangdong, China) as the basal diet, with crude protein ≥ 17%, crude fiber ≤ 8%, crude ash < 8%, calcium 0.40–1.20%, total phosphorus 0.40%, sodium chloride 0.30–0.80%, and lysine 0.90%. Ingredient and fatty acid contents of the basal diet are shown in Supplementary Tables S1, S2, respectively. GAA was mixed into the basal diet daily in proportion. The pigs were given free access to feed and water. Each pig was weighed at the beginning and end of the trial, and daily feed intake was recorded to evaluate the average daily gain (ADG), average daily feed intake (ADFI), and the feed to gain ratio (F/G).

### Slaughter and sample collection

After fasting for ~12 h, pigs were electro-stunned and exsanguinated according to the current slaughterhouse practices. The carcass weight was measured immediately post-mortem to calculate the dressing percentage. Samples (200 g) were obtained from the left *longissimus thoracis* (LT) between the 7th and 10th ribs, frozen in liquid nitrogen, and then stored at −80°C before performing measurements of chemical composition, oxidative stability, free amino acid contents, fatty acid contents, and gene expression. Back fat (BF) samples (100 g) at the location corresponding to LT were collected, frozen in liquid nitrogen, and then stored at −80°C before performing measurements of fatty acid profile and gene expression.

### Carcass traits and meat quality

Carcass traits and meat quality were measured as described previously (16). The average BF thickness was measured at the first rib, lumbar, and the last rib of the left side. The loin muscle area was measured by tracing the outline of the LT area at the 10th rib and then measuring the area using a digital planimeter (KP-90N, Koizumi, Japan) for each pig. Post-slaughter, the pH were measured by a pH meter (testo-205, Testo, Lenzkirch, Germany) with automatic temperature compensation, which was calibrated using pH 4.01 and 7.00 buffers. The meat color was measured using a colorimeter (CR-410, Minolta, Chiyoda, Japan) calibrated to the CIE LAB color system using a CR-A44 calibration plate (No.16433029). The illuminant was D65 source and the observer was standard 2°. For the determination of shear force at 24 h postmortem, a piece of about 200 g LT sample (each pig) was placed in a plastic bag and cooked to an internal temperature of 70°C in a 80°C water bath (HWS-28, 752 mm width × 345 mm depth × 210 mm height; Shanghai Yiheng Instrument Co., Ltd., China), and then cooled to room temperature. Ten cores (1.27 cm diameter × 10 cm length) parallel to muscle fiber were sheared perpendicular to the fiber direction using a digital muscle tenderness tester (C-LM3B, TENOVO, China). The average of 5 shear measurements was used as shear force for each core, and the shear force was expressed in newtons (N).

### Chemical composition and antioxidant status

Contents of moisture, crude protein, IMF, ash, and inosinic acid in LT were measured by freeze-dryer (ALPHA 2–4 LSC, Christ Martin GmbH), automatic nitrogen analyzer (8400, FOSS, Denmark), fat analyzer (2055 SOXTEC, FOSS, Denmark), box resistance furnace (SX2-4-10N, Yiheng, Shanghai, China), and high performance liquid chromatography (HPLC, LC-20AD, Shimazu, Japan), respectively, as described previously (16). Briefly, the contents of moisture, crude protein, IMF, and ash were analyzed by the freeze-drying method, Kjeldahl method, Soxhlet extraction, and burning method, respectively.

The sample preparation procedure comprised homogenization of LT (0.1 g) and 0.9% saline (0.9 mL), and 1,500 r/min for 2 min, followed by centrifugation at 3,500 × *g* for 15 min at 4°C to collect the supernatant. Then, contents of cholesterol, triglyceride, and malondialdehyde (MDA) and activities of total antioxidant capacity (T-AOC), glutathione peroxidase (GSH-Px), and superoxide dismutase (SOD) were determined according to the instructions (F001-1-1, F002-1-1, A003-2-2, A015-1-2, A005-1-2, A001-1-2; Nanjing Jiancheng Bioengineering Institute, Nanjing, China). The plate was read by a multi-functional enzyme labeling instrument (Spectra Max M5, Molecular Devices, USA) at 532 nm (MDA), 520 nm (T-AOC), 412 nm (GSH-Px), and 450 nm (SOD).

### Free amino acid contents in *Longissimus thoracis*

Freeze-dried LT samples (0.2 g) and 10% sulfosalicylic acid (1.5 mL) were homogenized for 15 min and then centrifuged at 12,000 × *g* for 15 min at 4°C to remove proteins. The supernatant was filtered with a 0.22-μm filter, and then the free amino acid contents were measured by an amino acid analyzer (L-8900, Hitachi Ltd., Tokyo, Japan) by the post-column derivatization of ninhydrin. Amino acids were expressed as mg/100 g LT based on the wet weight. Essential amino acids (EAA) included lysine, methionine, tryptophan, threonine, arginine, histidine, leucine, isoleucine, phenylalanine, and valine; non-essential amino acids (NEAA) = total amino acids (TAA)–EAA; total umami components (TUC) included aspartate and glutamate; total sweet components (TSC) included alanine, glycine, serine, and threonine; and total bitter components (TBC) included arginine, histidine, isoleucine, leucine, methionine, phenylalanine, and valine.

### Fatty acid contents in *Longissimus thoracis* and back fat

Fatty acids were analyzed with a gas chromatograph (6890, Agilent, California, USA) equipped with a flame ionization detector, as described previously (16). Each sample (10.0 g) was combined with 2.0 ml undecanoic acid, 1, 1′, 1″-(1,2,3-propanetriyl) ester solution, and hydrochloric acid at 70°C for 40 min. Then, 95% ethanol (10 mL) was added and the hydrolysate was extracted with petroleum ether mixture. Finally, saponification and methylation were performed to prepare fatty acid methyl esters. Fatty acid methyl esters were separated by TR-FAME GC columns (260M238P 100 m length × 0.25 mm diameter × 0.20 μm film thickness, Thermo Scientific, Massachusetts, USA). Fatty acid contents were based on peak areas of the internal standards, and identified from individual and mixed FAME standards (CRM47885, Sigma, Darmstadt, Germany). Saturated fatty acids (SFA) included C8:0, C10:0, C11:0, C12:0, C14:0, C15:0, C16:0, C17:0, C18:0, C20:0 and C22:0; monounsaturated fatty acids (MUFA) included C14:1, C16:1, C18:1n-9, C20:1, C22:1, and C24:1; and polyunsaturated fatty acids (PUFA) included C18:2n-6, C18:3n-3, C18:3n-6, C20:2, C20:3n-6, C20:4n-6, C20:3n-3, C22:2, and C22:6n-3.

### Gene expression

Total RNA was extracted from LT and BF samples with TRIzol reagent (Takara Biotechnology, Dalian, China). The qualified RNA samples were reverse-transcribed according to the manufacturer's protocol (RR047A, TaKaRa).

Real-time PCR primer sequences are shown in Supplementary Table S3. The relative expressions of genes were determined by qPCR analysis using SYBR Green mix (Bio-Rad, Hercules, CA, USA) on the CFX96 Real-Time PCR Detection System (Bio-Rad Laboratories). Real-time PCR was performed in the same manner, as described previously (17). β*-actin* was used to normalize the mRNA expression of each target gene, and the relative expression data were calculated using the 2^−ΔΔCt^ method (17), where ΔΔCt = (Ct_target_ – Ct_β − *actin*_)treatment – (Ct_target_ − *Ct*_β−*actin*_)control. The relative expressions of *adipose triglyceride lipase* (*ATGL*), *stearoyl CoA desaturase* (*SCD*), *fatty acid binding proteins* (*FABP*), *ankyrin* 1 (*ANK*1), *acetyl Co A carboxylase* (*ACC*), *peroxisome proliferator-activated receptor* γ *(PPAR*γ), *fatty acid synthase* (*FAS*), 2, 4*-dienoyl-Co A reductase* 1 (*DECR*1), *sterol regulatory element binding protein* (*SREBP*), and *malic enzyme* 1 (*ME*1) were determined.

### Statistical analysis

All data were computed using the Independent Samples *T*-Test in SPSS 25.0 (SPSS Inc., Chicago, IL, USA). For assessing the indicators, the pig was considered as the experimental unit. *P* ≤ 0.05 was considered significant, and 0.05 < *P* ≤ 0.10 was considered as a trend.

## Results

### Growth performance and carcass traits

GAA caused no significant difference in growth performance or carcass traits of Tibetan pigs (*P* > 0.05, Table 1).

**Table 1:** Effect of dietary GAA on growth performance and carcass traits of *Longissimus thoracis* in Tibetan pigs.

**Items**	**Control**	**GAA**	**SEM**	***P*-value**
**Growth performance**				
Initial weight (kg)	22.61	21.94	0.90	0.608
Final weight (kg)	54.33	53.28	1.74	0.689
ADG (g)	253.78	250.67	15.68	0.897
ADFI (kg)	1.43	1.33	0.04	0.105
F/G	5.82	5.35	0.28	0.263
**Carcass traits**				
Carcass weight (kg)	36.53	34.25	2.33	0.504
Carcass yield (%)	66.63	66.30	1.46	0.878
Abdominal fat weight (kg)	2.34	2.60	0.22	0.440
Back fat thickness (mm)	44.89	40.72	1.66	0.113
Loin muscle area (cm^2)^	18.34	18.31	1.02	0.982

ADG, average daily gain; ADFI, average daily feed intake; F/G, feed to gain ratio.

### Meat quality

Marbling score of LT tended to be higher in the GAA group than in the control group (*P* = 0.065, Table 2). No differences were observed in meat color, pH, drip loss, and shear force between the diets (*P* > 0.05).

**Table 2:** Effect of dietary GAA on meat quality of *Longissimus thoracis* in Tibetan pigs.

**Items**	**Control**	**GAA**	**SEM**	***P*-value**
L*_45min_	38.21	36.80	0.74	0.218
L*_24h_	46.11	44.55	1.82	0.561
a*_45min_	18.52	18.94	0.36	0.429
a*_24h_	19.66	20.66	0.65	0.299
b*_45min_	3.00	3.01	0.25	0.978
b*_24h_	6.12	6.77	0.78	0.573
pH _45min_	6.31	6.34	0.04	0.659
pH_24h_	5.65	5.61	0.07	0.733
Drip loss _24h_ (%)	2.28	2.36	0.19	0.795
Drip loss _48h_ (%)	3.05	2.67	0.28	0.395
Shear force (N)	53.41	52.90	2.65	0.898
Marbling score	2.24	2.92	0.23	0.065

### Chemical composition and antioxidant status of *Longissimus thoracis*

Dietary GAA had no effect on the content of moisture, crude protein, ash, and inosinic acid (*P* > 0.05, Table 3). However, dietary GAA tended to increase the IMF content (*P* = 0.091).

**Table 3:** Effect of dietary GAA on chemical composition and antioxidant status of *Longissimus thoracis* in Tibetan pigs.

**Items**	**Control**	**GAA**	**SEM**	***P*-value**
Chemical composition				
Moisture (%)	71.38	69.45	1.03	0.228
Crude protein (%)	23.46	23.33	0.48	0.854
Ash (%)	1.76	2.00	0.11	0.175
IMF (%)	3.65	6.39	1.03	0.091
Inosinic acid (mg/100 g)	305.33	328.17	20.06	0.461
Triglycerides (μmol/gprot)	57.70	58.65	6.74	0.923
Total cholesterol (μmol/gprot)	17.45	16.35	2.90	0.798
Antioxidant status				
MDA (nmol/mgprot)	0.14	0.09	0.00	<0.001
SOD (U/mgprot)	77.23	100.19	1.85	<0.001
T-AOC (U/mgprot)	0.21	0.22	0.01	0.218
GSH-Px (U/mgprot)	4.30	4.19	0.15	0.628

IMF, intramuscular fat; MDA, malondialdehyde; SOD, superoxide dismutase; T-AOC, total antioxidant capacity; GSH-Px, glutathione peroxidase.

Feeding GAA significantly decreased the MDA content but increased the SOD activity compared to the control group (*P* < 0.001). There was no significant difference in the T-AOC and GSH-Px activities between treatments (*P* > 0.05).

### Free amino acid contents of *Longissimus thoracis*

Feeding GAA increased the contents of glutamate (*P* = 0.002), glutamine (*P* = 0.018), TUC (*P* = 0.029), and ∑NEAA (*P* = 0.093) in pig LT, while it decreased the ratios of TBC/TAA (*P* = 0.049) and TSC/TAA (*P* = 0.040) compared to the control group (Table 4).

**Table 4:** Effect of dietary GAA on free amino acid contents of *Longissimus thoracis* in Tibetan pigs (mg/100 g based on wet weight).

**Items**	**Control**	**GAA**	**SEM**	***P*-value**
Valine	37.01	36.80	3.28	0.964
Isoleucine	13.09	14.08	1.39	0.628
Leucine	44.68	46.47	3.78	0.744
Threonine	20.05	21.99	2.08	0.525
Lysine	37.55	41.44	5.01	0.596
Histidine	16.82	19.67	1.89	0.311
Arginine	15.89	18.99	2.97	0.989
Phenylalanine	23.63	25.13	2.39	0.668
Taurine	169.71	230.91	29.34	0.182
Aspartic acid	4.44	3.00	1.07	0.370
Serine	25.95	28.34	3.06	0.601
Asparagine	5.48	5.37	0.69	0.916
Glutamate	10.58	15.78	0.88	0.002
Glutamine	95.17	141.34	11.58	0.018
Glycine	31.29	34.11	2.92	0.511
Alanine	157.86	182.31	13.59	0.235
Tyrosine	25.91	25.96	2.45	0.988
Ornithine	3.38	4.86	1.83	0.854
Proline	23.40	25.06	2.31	0.627
Anserine	112.96	155.23	23.62	0.240
Carnosine	5963.44	6697.46	518.55	0.346
∑EAA	208.72	221.38	21.96	0.692
∑NEAA	578.86	724.30	54.39	0.093
TAA	787.58	945.68	73.55	0.164
TUC	13.54	17.27	1.00	0.029
TBC	151.11	157.96	15.35	0.759
TSC	235.15	266.74	19.47	0.279
Ratio				
TUC/TAA	0.02	0.02	0.00	1.000
TBC/TAA	0.19	0.17	0.01	0.049
TSC/TAA	0.30	0.28	0.01	0.040

EAA, essential amino acid; NEAA, nonessential amino acids; TAA, total amino acids; TUC, total umami components; TSC, total sweet components; TBC, total bitter components.

### Fatty acid contents/profile of *Longissimus thoracis*

As shown in Table 5, compared to the control group, dietary GAA significantly increased the contents of C24:0, C20:3n-6, C20:4n-6, and ∑PUFA in LT (*P* < 0.05), and it tended to increase the contents of C14:0, C16:1, C18:1n-9, C18:2n-6, C18:3n-3, C20:2, total unsaturated fatty acids (UFA), and ∑MUFA (0.05 < *P* ≤ 0.10). However, as shown in Supplementary Table S4, the fatty acid profile of LT was not altered by GAA.

**Table 5:** Effect of dietary GAA on fatty acid contents of *Longissimus thoracis* in Tibetan pigs (based on wet weight).

**Items**	**Control**	**GAA**	**SEM**	***P*-value**
**FA (mg/100 g)**				
C10:0	5.69	7.92	1.07	0.210
C12:0	4.03	6.57	1.68	0.312
C14:0	56.84	105.58	18.82	0.097
C16:0	988.56	1765.22	304.97	0.102
C17:0	5.05	8.07	1.51	0.193
C18:0	400.79	679.84	119.40	0.129
C20:0	9.89	15.95	2.72	0.148
C24:0	8.45	12.97	0.95	0.009
C16:1	162.22	314.16	51.78	0.068
C18:1n-9t	5.29	8.69	1.61	0.208
C18:1n-9	1538.19	2684.85	432.94	0.091
C20:1	33.22	57.88	9.74	0.106
C18:2n-6	220.40	352.24	42.89	0.056
C18:3n-3	6.85	10.48	1.37	0.092
C20:2	10.57	17.80	2.49	0.069
C20:3n-6	5.95	9.10	0.68	0.010
C20:4n-6	33.01	43.94	1.88	0.004
**Category (g/100 g)**				
∑FA	3.49	6.11	0.99	0.091
∑SFA	1.47	2.60	0.45	0.106
∑UFA	2.01	3.50	0.54	0.080
∑MUFA	1.74	3.07	0.49	0.087
∑PUFA	0.28	0.44	0.05	0.043
**Ratio**				
MUFA/SFA	1.20	1.20	0.02	0.927
PUFA/SFA	0.22	0.18	0.02	0.351
UFA/SFA	1.41	1.38	0.04	0.627
n-6/n-3	39.21	40.13	2.87	0.837

FA, fatty acids; SFA, saturated fatty acids; UFA, unsaturated fatty acids; MUFA, monounsaturated fatty acids; PUFA, polyunsaturated fatty acids.

### Fatty acid contents/profile of back fat

The fatty acid contents of BF are shown in Table 6. Dietary GAA reduced the contents of C10:0, C12:0, C14:0, and C16:0 in BF of Tibetan pigs (*P* < 0.05), increased the contents of C22:0, C20:1, C22:1, C24:1, C20:2, C20:3n-3, C22:2, and PUFA/SFA ratio (*P* < 0.05), and tended to increase C20:0 content and the ratios of MUFA/SFA and UFA/SFA (0.05 < *P* ≤ 0.10). When fatty acid profile was expressed as % total fatty acids (Supplementary Table S5), dietary GAA decreased the abundances of most of the fatty acids, such as C8:0, C20:0, C22:0, C18:1n-9, C20:1, C22:1, C18:2n-6, C20:2, C20:3n-3, C22:2, and ∑PUFA (*P* < 0.05).

**Table 6:** Effect of dietary GAA on fatty acid contents of back fat in Tibetan pigs (based on wet weight).

**Items**	**Control**	**GAA**	**SEM**	***P*-value**
**FA (mg/100 g)**				
C8:0	6.58	7.12	0.19	0.134
C10:0	58.02	50.87	2.16	0.043
C11:0	252.25	217.25	15.60	0.254
C12:0	75.98	63.27	2.17	0.015
C14:0	1396.98	1115.17	48.48	0.009
C15:0	35.95	34.17	1.22	0.331
C16:0	22720.97	20299.62	603.30	0.018
C17:0	183.43	197.77	8.30	0.383
C18:0	10140.60	9955.45	628.09	0.865
C20:0	215.95	263.55	13.38	0.091
C22:0	13.25	20.35	1.22	0.003
C14:1	19.28	15.15	2.25	0.229
C16:1	2231.77	1867.53	199.31	0.255
C18:1n-9	32469.07	32202.90	673.63	0.787
C20:1	950.83	1116.87	35.48	0.009
C22:1	16.35	20.77	0.46	<0.001
C24:1	4.65	5.28	0.13	0.025
C18:2n-6	9369.05	9744.38	277.12	0.361
C18:3n-3	328.43	335.22	11.49	0.687
C18:3n-6	18.72	18.25	1.54	0.836
C20:2	534.53	641.25	6.77	<0.001
C20:3n-6	93.13	86.47	6.81	0.511
C20:4n-6	161.13	172.32	9.43	0.424
C20:3n-3	78.97	91.12	1.99	0.002
C22:2	9.45	11.45	0.31	0.004
C22:6n-3	6.10	7.18	0.93	0.430
**Category (g/100 g)**				
∑FA	81.12	78.35	2.10	0.375
∑SFA	35.10	32.22	1.24	0.146
∑UFA	46.29	46.34	0.97	0.973
∑MUFA	35.69	35.23	0.72	0.662
∑PUFA	10.60	11.11	0.30	0.255
**Ratio**				
MUFA/SFA	1.02	1.10	0.02	0.084
PUFA/SFA	0.30	0.35	0.01	0.018
UFA/SFA	1.32	1.45	0.03	0.059
n-6/n-3	23.10	23.33	0.21	0.493

FA, fatty acids; SFA, saturated fatty acids; UFA, unsaturated fatty acids; MUFA, monounsaturated fatty acids; PUFA, polyunsaturated fatty acids.

### Expression of lipid metabolism-related genes in *Longissimus thoracis* and back fat

The effects of dietary GAA on the mRNA levels of genes related to lipid metabolism are reported in Table 7. Transcription of *ATGL* (*P* = 0.063), *SCD* (*P* = 0.036), and *FAS* (*P* = 0.025) in LT was increased by GAA treatment compared to the control group. No differences were found in the expressions of *FABP, ANK*1, *ACC, PPAR*γ, *DECR*1, *SREBP*, or *ME1* in LT between the two groups (*P* > 0.05).

**Table 7:** Effect of dietary GAA on expression of lipid metabolism-related genes in *Longissimus thoracis* and back fat of Tibetan pigs.

**Items**	**Control**	**GAA**	**SEM**	***P*-value**
***Longissimus thoracis***				
*ATGL*	1.07	1.75	0.23	0.063
*SCD*	1.09	1.92	0.24	0.036
*FABP*	1.05	0.85	0.12	0.304
*ANK1*	1.07	0.86	0.14	0.335
*ACC*	1.02	1.13	0.06	0.225
*PPARγ*	1.03	0.82	0.10	0.165
*FAS*	1.05	1.64	0.16	0.025
*DECR1*	1.07	0.98	0.13	0.670
*SREBP*	1.04	1.04	0.13	0.997
*ME1*	1.08	0.87	0.14	0.334
**Back fat**				
*ATGL*	1.05	2.28	0.12	<0.001
*SCD*	1.10	1.28	0.25	0.612
*FABP*	1.08	0.88	0.19	0.504
*ANK1*	1.12	1.16	0.26	0.909
*ACC*	1.07	1.21	0.17	0.585
*PPARr*	1.01	0.64	0.09	0.026
*FAS*	1.06	1.25	0.19	0.472
*DECR1*	1.03	0.92	0.14	0.580
*SREBP*	1.12	1.39	0.22	0.413
*ME1*	1.10	0.65	0.14	0.093

ATGL, adipose triglyceride lipase; SCD, stearoyl CoA desaturase; *FABP*, fatty acid binding proteins; ANK1, ankyrin 1; ACC, acetyl Co A carboxylase; *PPAR*γ, peroxisome proliferator-activated receptor γ; FAS, fatty acid synthase; DECR1, 2, 4-dienoyl-Co A reductase 1; *SREBP*, sterol regulatory element binding protein; ME1, malicenzyme 1.

In BF, dietary GAA increased *ATGL* expression (*P* < 0.001), but it reduced the expressions of *PPARr* (*P* = 0.026) and *ME1* (*P* = 0.093). No significant difference was observed in the expression of other genes related to lipid metabolism between treatments (*P* > 0.05).

## Discussion

Our results showed that dietary supplementation of GAA did not alter the growth performance of Tibetan pigs, which is consistent with the study by McBreairty et al. (18), who reported that there was no difference in the body weight of Yucatan miniature pigs among the GAA, creatine, and basic diet groups. However, some studies have suggested that GAA can improve the pig growth performance. For example, GAA supplementation increased ADG and ADFI in crossbred pigs (10). Body weight, ADG, and feed conversion rate increased quadratically in pigs with increasing dietary GAA (7). In addition, feeding time had different effects on growth performance at the same supplemental dose. One report stated that final body weight and ADG were higher in pigs fed GAA for 60 days than in pigs fed for 25 days (7). However, our experiment lasted for 125 days, and dietary GAA failed to improve the growth performance of Tibetan pigs. This discrepancy might be due to the differences in genotypes and physiological stages of Tibetan pigs and crossbred pigs (Duroc × Landrace × Yorkshire). Tibetan pigs are slow-growing breeds with more fat deposits (15), which makes them different from lean crossbred pigs (19).

Previous studies have shown that GAA supplementation improves lean meat production in pigs (6, 7, 11). These studies suggest that GAA improves creatine reserve and energy metabolism in pig muscle to enhance lean meat production (6, 20, 21). However, GAA did not improve the carcass parameters in Tibetan pigs. Thus, the effect of GAA on carcass parameters in Tibetan pigs may be related to the pig breed. Long-term selection in commercial pigs (PIC, Duroc × Landrace × Yorkshire) has resulted in enhanced reproductive and growth capabilities, higher lean carcass percentages, and more efficient muscle growth (22). The potential for protein deposition in Tibetan pigs is low (2), and it can be speculated that GAA has a limited effect on the protein deposition capacity of Tibetan pigs.

Meat quality, which includes traits such as, color, pH, drip loss, and shear force, is one of the most important economic characteristics of the pig industry. In this study, the addition of GAA did not cause a significant difference in the meat quality of Tibetan pigs, which was similar to the study by Jayaraman et al. (7). Marbling score is an important indicator of IMF (23). In our study, the effect of GAA on the marbling score and IMF was the same, with a trend toward improvement. GAA supplementation increased the IMF content in LT of Tibetan pigs by 75.12%, which was similar to previous studies showing that the IMF content was significantly increased in pigs and fish fed GAA (10, 24). In addition, GAA did not alter the protein nutrition of Tibetan pigs, which was consistent with previous studies (5, 25) claiming that GAA had no significant effect on muscle chemical composition.

Lipid peroxidation significantly affected the meat quality (26). MDA is a secondary product of lipid oxidation and a major indicator of oxidative stress (27). SOD plays an important role in antioxidant defense (28). Antioxidant enzymes can protect the lipids from oxidative damage. GAA indirectly acts as an antioxidant due to the antioxidant capacity of creatine and arginine metabolites (29). Supplementation of GAA in Tibetan pigs beneficially increased the SOD activity and decreased the MDA content in LT, suggesting that GAA enhanced the antioxidant capacity of LT. These findings are consistent with those presented by Li et al. (30), who reported an increase in serum SOD, glutathione, and glutathione/oxidized glutathione ratio in the GAA group compared to the control group. GAA supplementation in pig diets significantly reduced the muscle MDA content and increased the GSH-Px and SOD activities (31). GAA supplementation increased breast T-AOC activity and decreased the levels of reactive oxygen species and MDA in broiler chickens (32). Therefore, GAA diet improved the antioxidant status of LT in Tibetan pigs, prolonging the shelf life of meat. Consumption of meat with high antioxidant capacity is beneficial to consumers' health.

Free amino acids in muscle participate in many reactions that affect the flavor and play a key role in meat flavor (33). Amino acids are usually divided into sweet amino acids, bitter amino acids, and umami amino acids (34). Glutamate and aspartate can synergize with inosinic acid to enhance the flavor and buffer undesired flavors, such as acids and bases (35). Glutamate and TUC contents of LT in the GAA group were higher than those in the control group, indicating that pork umami was altered by GAA. The levels of most of the free amino acids in LT were numerically higher in the GAA group than in the control group, and these free amino acids and reducing sugars in the muscles can produce flavor substances. The proportions of bitter and sweet amino acids were decreased in the GAA group. The reduction of bitter amino acids may improve the meat flavor to a certain extent. However, it is unclear whether pork flavor is altered by reducing the sweet amino acid content. It might be necessary to conduct a meat taste test to distinguish the flavor changes of pork.

Fatty acids in muscle play an important role in the nutritional value, flavor, and oxidative stability of meat. Tu et al. (36) reported that C14:0, C17:0, C20:0, and C22:0 were the main fatty acids in pork. However, Choi et al. (37) showed that the major fatty acids in pork were C16:0 and C18:0. These results were not exactly the same as in our study. The most abundant fatty acids in the muscle of Tibetan pigs were C18:1n-9, C16:0, C18:0, and C18:2n-6. The fatty acid profile of pork is highly affected by dietary fat sources (38). This might be one of the reasons for the difference in the above-described results. Generally, the unbalanced ratio of SFA and UFA, and n3/n6 affect the health of humans, while PUFA is beneficial to human growth and development. n-3 and n-6 PUFA help to improve long-term glycemic control and insulin secretion capacity and reduce cardiovascular metabolic syndrome and obesity rates in the population (39–41). The Tibetan pig is a type of a local fattening breed, which is characterized by strong fat production potential and high UFA content. GAA reduced most of the SFA content and increased most of the UFA content in BF. Importantly, consumers eat commercial meat, which combines lean meat and subcutaneous fat. Therefore, an improvement in fatty acid composition of LT and BF is beneficial to consumer health. The fatty acid profile of diets was the same. Animals cannot synthesize C18:2n-6 and C18:3n-3 (41), which are derived entirely from the diet. The increasing trend in C18:2n-6 and C18:3n-3of LT in the GAA group may be because GAA improves the transport and deposition efficiency of these fatty acids in LT, which are beneficial to human health (42, 43). There are concerns that high intake of n-6 PUFA will lead to excessive chronic inflammation. However, studies have found that increasing the intake of n-6 PUFA did not increase the contents of many inflammation markers (39, 42, 44). Studies demonstrated that 48% of the 175 countries (47 developed and 128 developing countries) have an C20:4n-6 intake of < 150 mg/day (45), and the intake of n-3 and n-6 PUFA in diet of lactating women, adolescents, and the elderly is insufficient (46). High PUFA content in pork treated with GAA might be suitable for people from specific populations and regions. It is worth mentioning that further processing and cooking treatment can change the fatty acid composition of fresh meat to a great extent (47, 48). In addition, the n6/n3 ratio (40) was high in LT, mainly because of the high n6/n3 ratio (16) in the basal diet. Commercial diet-fed Tibetan pigs have a higher n6/n3 ratio (31–43) in LT (49). Tibetan pigs are generally reared through grazing, and the n6/n3 ratio in LT of Tibetan pigs that consume forage is <5 (50). It has also reported that the n6/n3 ratio is about 17 (51). GAA should be added under grazing conditions to verify whether GAA changes the LT fatty acid profile of Tibetan pigs.

Fatty acids can directly contribute to the formation of pork flavor, and UFA (C18:1n-9, C18:1n-9t, C17:1, C18:2 n-6, and C16:1) and C18:0 play an important role in the formation of subsequent flavor products (52). The increase in the contents of these flavor precursors in our study contributes to the desirable flavor attributes of Tibetan pork, which can improve the eating experience of consumers. Furthermore, there is a positive correlation between the PUFA content and the nutritional value of meat, which is beneficial. However, PUFA is prone to oxidation, leading to myoglobin oxidation, discoloration, softening, and rancidity of pork (53). In this study, the increase in PUFA in LT of pigs fed GAA did not have a negative impact on pork color or meat quality. This may be attributed to the protection of PUFA by the antioxidant capacity of GAA (GAA increased the SOD activity and reduced the MDA content in LT).

Our study showed that dietary GAA had a significant effect on the fatty acid contents of LT and BF. PUFA levels in pig tissue only depend on the diet. Thus, markedly elevated levels of individual PUFA found in the BF and LT of Tibetan pigs could be explained by the fact that GAA enhances the ability of Tibetan pigs to store dietary unsaturated lipids in their tissues. The effect of GAA on fatty acid profiles in muscle and adipose tissue of pigs occurs in a tissue-specific manner. GAA increased the PUFA contents in LT, while it decreased most of the fatty acid contents in BF. Considering the fatty acid profile, GAA did not alter the fatty acid composition of LT, but it decreased the abundance of most of the fatty acids in BF. This can be explained as follows: adipocytes are the predominant cell type in BF and myofibers are the predominant cell type in LT (54). Intramuscular adipocytes use glucose as the main carbon source of fatty acids, while subcutaneous adipocytes use acetate (55).

The concentration of fatty acids in tissue is mainly regulated by key genes encoding specific metabolic enzymes related to lipid metabolism, which affects the deposition of IMF and marbling score in muscle. *FAS* and *ACC* are the key regulatory enzymes in fatty acid synthesis (56). *SCD* is responsible for converting SFA into MUFA and PUFA (57, 58). In our study, pigs receiving GAA supplementation exhibited higher expression levels of *FAS* and *SCD* (56.19% and 76.15%, respectively), which indicated higher fatty acid synthesis in LT than that in control-fed pigs. The results were consistent with those of fatty acids in LT. Therefore, higher levels of IMF, MUFA, and PUFA in LT of pigs in the GAA group may be due to the up-regulation of mRNA expression levels of adipogenesis-related genes (*FAS* and *SCD*), and GAA-induced IMF deposition may be related to the promotion of adipogenesis capacity in growing muscles.

*PPAR*γ and *ME1* are rate-limiting enzymes for fatty acid synthesis. *ME1* supplies nicotinamide adenine dinucleotide phosphate and acetyl-CoA required for fatty acid biosynthesis (59). *PPAR*γ is the main regulator of adipogenesis, which can promote adipocyte differentiation and fat deposition (60). *ATGL* is an important enzyme that releases fatty acids from triacylglycerol stores (61). In the present study, GAA significantly down-regulated the mRNA levels of *PPAR*γ and *ME1* but up-regulated the mRNA level of *ATGL* in BF, suggesting that GAA may accelerate the breakdown of fatty acids and reduce fat synthesis in BF. *FABP* and *SREBP* are involved in fat metabolism, and *SREBP* may promote *PPAR*γ transcription to regulate fatty acid biosynthesis (56, 62). GAA did not affect the expression of *FABP* and *SREBP* in LT or BF, but it decreased *PPAR*γ expression in BF. The above results suggest that there are different mechanisms of lipid metabolism in BF and LT. All these findings suggested that the improvement in the fatty acid contents and expression levels of lipid metabolism-related genes in LT and BF are differentially regulated.

GAA supplementation can reduce the content of arginine used to synthesize creatine and increase the content of available arginine. Guo et al. (13) reported that arginine supplementation increased the C14:0, C16:0, C16:1, C18:0, C18:1, C18:2n-6, C18:3n-3, C20:0, C20:1c9 and C20:2, total fatty acids, SFA, MUFA, and PUFA levels in pigs. Previous studies have also shown that adding arginine can increase the IMF content and reduce the accumulation of systemic fat in pigs (63, 64). The findings of arginine studies are consistent with the performance of GAA supplementation in our study. Arginine differentially regulates the expression of lipid metabolism-related genes in skeletal muscle and subcutaneous fat, which is conducive to fat production in muscle and fat decomposition in adipose tissue (65). The effect of GAA on lipid metabolism in Tibetan pigs may be achieved by increasing the available arginine.

## Conclusions

In conclusion, the results of this study showed that dietary GAA had no significant effect on growth performance, carcass parameters, or meat quality of Tibetan pigs, but it increased the contents of IMF and umami amino acids in LT and altered the fatty acid contents in LT and BF. In addition, GAA up-regulated the expression of lipogenic genes in LT and promoted the expression of lipolytic genes, and it decreased the expression of lipogenic genes in BF. These findings will provide a basis for the production of high-quality Tibetan pork.

## Data availability statement

The original contributions presented in the study are included in the article/Supplementary material, further inquiries can be directed to the corresponding author/s.

## Ethics statement

The animal study was reviewed and approved by Animal Care and Use Committee of Guangdong Academy of Agricultural Sciences.

## Author contributions

YC and XM conceived the project, designed the experiments, and drafted the manuscript. ZT, ZL, and TR performed the experiments. YC and ZT analyzed the data. YC, XM, and MY revised the manuscript. All authors have read and approved the final manuscript.

## Funding

This project has received funding from the Provincial Agricultural Science and Technology Innovation Promotion and Agricultural Resources and Ecological Environmental Protection Construction Project (2021KJ266), Agricultural Competitive Industry Discipline Team Building Project of Guangdong Academy of Agricultural Sciences (202118TD), Guangdong Modern Agro-industry Technology Research System (2022KJ115), Special fund for Scientific Innovation Strategy-Construction of High Level Academy of Agriculture Science-Distinguished Scholar (R2020PY-JC001, R2020YJYB2002), and Start-up Research Project of Maoming Laboratory (2021TDQD002).

## Conflict of interest

The authors declare that the research was conducted in the absence of any commercial or financial relationships that could be construed as a potential conflict of interest.

## Publisher's note

All claims expressed in this article are solely those of the authors and do not necessarily represent those of their affiliated organizations, or those of the publisher, the editors and the reviewers. Any product that may be evaluated in this article, or claim that may be made by its manufacturer, is not guaranteed or endorsed by the publisher.
