# Supplementary material for: Effect of guanidine acetic acid on meat quality, muscle amino acids, and fatty acids in Tibetan pigs
Source: Front Vet Sci. 2022 Oct 11;9:998956. doi: 10.3389/fvets.2022.998956 (PMC9592698; doi:10.3389/fvets.2022.998956)
Supplement: Supplementary file 1 [file Data_Sheet_1.docx]

Supplementary Material

**Table S1** Ingredient contents of the basal diet (%, as-fed basis).

| **Ingredients** | **Content** |
| --- | --- |
| Corn | 52 |
| Sorghum | 8 |
| Soybean meal | 20 |
| Distillers Dried Grains with Solubles | 4 |
| Rice bran meal | 4 |
| Corn gluten feed | 3 |
| Wheat middlings | 5 |
| Premix | 4 |
| Total | 100 |

**Table S2** Fatty acid contents / profile of the basal diet.

| **Items** | **Content (g/100g of diet)** | **Profile (% total fatty acids)** |
| --- | --- | --- |
| C14:0 | 0.012 | 0.431 |
| C15:0 | 0.003 | 0.091 |
| C16:0 | 0.543 | 19.239 |
| C16:1 | 0.022 | 0.779 |
| C17:0 | 0.006 | 0.202 |
| C18:0 | 0.093 | 3.307 |
| C18:1n-9 | 0.766 | 27.132 |
| C18:2n-6 | 1.259 | 44.614 |
| C18:3n-3 | 0.079 | 2.788 |
| C20:0 | 0.010 | 0.362 |
| C20:1 | 0.011 | 0.401 |
| C20:2 | 0.003 | 0.103 |
| C20:4n-6 | 0.003 | 0.106 |
| C22:0 | 0.005 | 0.192 |
| C24:0 | 0.007 | 0.255 |
| ∑SFA | 0.680 | 24.078 |
| ∑MUFA | 0.777 | 27.533 |
| ∑PUFA | 1.344 | 47.610 |

SFA, saturated fatty acids, MUFA, monounsaturated fatty acids; PUFA, polyunsaturated fatty acids.

**Table S3** Primers used for real-time PCR analysis.

| **Gene** | **Primer sequence (5'→3')** | **Size (bp)** | **Accession No.** |
| --- | --- | --- | --- |
| *ATGL* | F: AGTTCAGCCTGCGCAACCTC | 220 | EF 583921 |
|  | R: AGGGCACCATCATGGCTG |  |  |
| *SCD* | F: TCTGGGCGTTTGCCTACTATCT | 280 | AY 487829 |
|  | R: TCTTTGACGGCTGGGTGTTT |  |  |
| *FABP* | F: CAGGAAAGTCAAGAGCACCA | 227 | AJ 416020 |
|  | R: TCGGGACAATACATCCAACA |  |  |
| *ANK1* | F: GCCTTCACCCAGACCTGATA | 89 | XM 001928667.1 |
|  | R: GGCTTGAGATCACTGCTTCC |  |  |
| *ACC* | F: AAGACGGGGTCCTCATCTCC | 149 | NM 214379 |
|  | R: CGCCAGGTCGCTGTCATCT |  |  |
| *PPARγ* | F: AAGACGGGGTCCTCATCTCC | 149 | NM 214379 |
|  | R: CGCCAGGTCGCTGTCATCT |  |  |
| *FAS* | F: AGCCTAACTCCTCGCTGCAAT | 196 | AY 183428 |
|  | R: TCCTTGGAACCGTCTGTGTTC |  |  |
| *DECR1* | F: CCGAGACTGGGTCAGGTTT | 165 | NM 001190232.2 |
|  | R:TTGGGTCAAGACGGCTAAA |  |  |
| *SREBP* | F: GCGACGGTGCCTCTGGTAGT | 218 | AF 102873 |
|  | R: CGCAAGACGGCGGATTTA |  |  |
| *ME1* | F: ATCCAACCAGCAAAGCAGA | 176 | XM 001924333.4 |
|  | R: CACAACACCAAGGGCAACT |  |  |
| *β-actin* | F: CACGCCATCCTGCGTCTGGA | 380 | XM 003124280.4 |
|  | R: AGCACCGTGTGGCGTAGAG |  |  |

Note: F: forward; R: reverse

**Table S4** Effect of dietary GAA on fatty acid profile of *longissimus thoracis* in Tibetan pigs (% total fatty acids).

| **Items** | **Control** | **GAA** | **SEM** | ***P*-value** |
| --- | --- | --- | --- | --- |
| C10:0 | 0.15 | 0.13 | 0.01 | 0.260 |
| C12:0 | 0.11 | 0.11 | 0.00 | 0.513 |
| C14:0 | 1.59 | 1.70 | 0.06 | 0.248 |
| C16:0 | 27.91 | 28.50 | 0.53 | 0.444 |
| C17:0 | 0.15 | 0.14 | 0.01 | 0.403 |
| C18:0 | 11.29 | 11.02 | 0.36 | 0.614 |
| C20:0 | 0.28 | 0.27 | 0.02 | 0.451 |
| C24:0 | 0.28 | 0.25 | 0.05 | 0.585 |
| C16:1 | 4.79 | 5.03 | 0.30 | 0.578 |
| C18:1n-9t | 0.12 | 0.14 | 0.01 | 0.278 |
| C18:1n-9 | 43.83 | 44.15 | 0.33 | 0.515 |
| C20:1 | 0.93 | 0.96 | 0.04 | 0.711 |
| C18:2n-6 | 6.90 | 6.04 | 0.55 | 0.321 |
| C18:3n-3 | 0.23 | 0.18 | 0.03 | 0.367 |
| C20:2 | 0.32 | 0.30 | 0.02 | 0.626 |
| C20:3n-6 | 0.19 | 0.16 | 0.02 | 0.359 |
| C20:4n-6 | 1.17 | 0.85 | 0.21 | 0.337 |
| ∑SFA | 41.57 | 42.12 | 0.77 | 0.623 |
| ∑UFA | 58.44 | 57.88 | 0.77 | 0.623 |
| ∑MUFA | 49.63 | 50.31 | 0.26 | 0.109 |
| ∑PUFA | 8.81 | 7.57 | 0.80 | 0.325 |
| MUFA/SFA | 1.20 | 1.20 | 0.02 | 0.927 |
| PUFA/SFA | 0.22 | 0.18 | 0.02 | 0.351 |
| UFA/SFA | 1.41 | 1.38 | 0.04 | 0.628 |

SFA, saturated fatty acids; UFA, unsaturated fatty acids; MUFA, monounsaturated fatty acids; PUFA, polyunsaturated fatty acids.

**Table S5** Effect of dietary GAA on fatty acid profile of back fat in Tibetan pigs (% total fatty acids).

| **Items** | **Control** | **GAA** | **SEM** | ***P*-value** |
| --- | --- | --- | --- | --- |
| C8:0 | 0.009 | 0.008 | 0.000 | 0.042 |
| C10:0 | 0.065 | 0.071 | 0.001 | 0.019 |
| C11:0 | 0.279 | 0.309 | 0.020 | 0.359 |
| C12:0 | 0.081 | 0.094 | 0.003 | 0.010 |
| C14:0 | 1.433 | 1.717 | 0.062 | 0.009 |
| C15:0 | 0.044 | 0.044 | 0.001 | 0.788 |
| C16:0 | 25.911 | 27.991 | 0.074 | <0.001 |
| C17:0 | 0.252 | 0.227 | 0.010 | 0.149 |
| C18:0 | 12.598 | 12.505 | 0.567 | 0.926 |
| C20:0 | 0.334 | 0.267 | 0.015 | 0.014 |
| C22:0 | 0.026 | 0.016 | 0.001 | <0.001 |
| C14:1 | 0.020 | 0.024 | 0.003 | 0.375 |
| C16:1 | 2.405 | 2.740 | 0.237 | 0.408 |
| C18:1n-9 | 41.149 | 40.036 | 0.254 | 0.013 |
| C20:1 | 1.438 | 1.173 | 0.061 | 0.020 |
| C22:1 | 0.027 | 0.020 | 0.001 | <0.001 |
| C24:1 | 0.007 | 0.006 | 0.000 | 0.093 |
| C18:2n-6 | 12.458 | 11.549 | 0.269 | 0.038 |
| C18:3n-3 | 0.428 | 0.404 | 0.009 | 0.097 |
| C18:3n-6 | 0.023 | 0.023 | 0.001 | 0.938 |
| C20:2 | 0.824 | 0.660 | 0.023 | 0.003 |
| C20:3n-6 | 0.110 | 0.114 | 0.006 | 0.658 |
| C20:4n-6 | 0.219 | 0.198 | 0.007 | 0.059 |
| C20:3n-3 | 0.118 | 0.098 | 0.004 | 0.025 |
| C22:2 | 0.015 | 0.012 | 0.001 | 0.023 |
| C22:6n-3 | 0.009 | 0.007 | 0.001 | 0.205 |
| ∑SFA | 41.031 | 43.249 | 0.550 | 0.059 |
| ∑UFA | 59.248 | 57.060 | 0.560 | 0.063 |
| ∑MUFA | 45.045 | 43.995 | 0.378 | 0.163 |
| ∑PUFA | 14.203 | 13.065 | 0.283 | 0.018 |
| MUFA/SFA | 1.102 | 1.017 | 0.022 | 0.084 |
| PUFA/SFA | 0.348 | 0.302 | 0.011 | 0.020 |
| UFA/SFA | 1.450 | 1.320 | 0.032 | 0.061 |

SFA, saturated fatty acids; UFA, unsaturated fatty acids; MUFA, monounsaturated fatty acids; PUFA, polyunsaturated fatty acids.
